# The influence of economic policies on social environments and mental health 

**DOI:** 10.2471/BLT.23.290286

**Published:** 2024-01-31

**Authors:** Jo-An Occhipinti, Adam Skinner, P Murali Doraiswamy, Shekhar Saxena, Harris A Eyre, William Hynes, Patricia Geli, Dilip V Jeste, Carol Graham, Christine Song, Ante Prodan, Goran Ujdur, John Buchanan, Sebastian Rosenberg, Paul Crosland, Ian B Hickie

**Affiliations:** a The Brain and Mind Centre, Faculty of Medicine and Health, University of Sydney, 94 Mallet Street, Camperdown, New South Wales 2050, Australia.; b Department of Psychiatry and Behavioral Sciences, Duke University School of Medicine, Durham, United States of America (USA).; c Department of Global Health and Population, Harvard TH Chan School of Public Health, Boston, USA.; d Baker Institute for Public Policy, Rice University, Houston, USA.; e World Bank, Paris, France.; f Reform for Resilience Commission, Harvard T.H. Chan School of Public Health, Boston, USA.; g Global Research Network on Social Determinants of Mental Health and Exposomics, San Diego, USA.; h Brookings Institution, Washington, DC, USA.; i School of Computer, Data and Mathematical Sciences, Western Sydney University, Sydney, Australia.; j Business School, University of Sydney, Sydney, Australia.

## Abstract

Despite increased advocacy and investments in mental health systems globally, there has been limited progress in reducing mental disorder prevalence. In this paper, we argue that meaningful advancements in population mental health necessitate addressing the fundamental sources of shared distress. Using a systems perspective, economic structures and policies are identified as the potential cause of causes of mental ill-health. Neoliberal ideologies, prioritizing economic optimization and continuous growth, contribute to the promotion of individualism, job insecurity, increasing demands on workers, parental stress, social disconnection and a broad range of manifestations well-recognized to erode mental health. We emphasize the need for mental health researchers and advocates to increasingly engage with the economic policy discourse to draw attention to mental health and well-being implications. We call for a shift towards a well-being economy to better align commercial interests with collective well-being and social prosperity. The involvement of individuals with lived mental ill-health experiences, practitioners and researchers is needed to mobilize communities for change and influence economic policies to safeguard well-being. Additionally, we call for the establishment of national mental wealth observatories to inform coordinated health, social and economic policies and realize the transition to a more sustainable well-being economy that offers promise for progress on population mental health outcomes.

## Résumé

Malgré une meilleure sensibilisation et des investissements accrus dans les systèmes de santé mentale à travers le monde, les progrès en matière de réduction du degré de prévalence des troubles mentaux demeurent très limités. Dans le présent document, nous estimons que, pour réaliser des avancées au niveau de la santé mentale des populations, il est impératif de s'attaquer aux sources de cette détresse collective. En adoptant une perspective systémique, force est de constater que les politiques et structures économiques constituent les causes potentielles d'une mauvaise santé mentale. Les idéologies néolibérales, qui privilégient l'optimisation économique et la croissance ininterrompue, contribuent à promouvoir l'individualisme, l'insécurité professionnelle, la pression pesant sur les travailleurs, le stress parental, l'isolement social et un large éventail de facteurs associés à une dégradation de la santé mentale. Nous insistons sur la nécessité de faire appel à des chercheurs et défenseurs actifs dans ce domaine, afin de jouer un rôle dans la politique économique en attirant l'attention sur les implications pour le bien-être et la santé mentale. Nous plaidons pour une transition vers une économie du bien-être visant à rapprocher les intérêts commerciaux de la prospérité sociale et collective. L'intervention de personnes ayant été confrontées à des troubles mentaux, de praticiens et de chercheurs est nécessaire pour mobiliser les communautés en faveur d'un changement et influencer les politiques économiques pour préserver le bien-être. Par ailleurs, nous militons pour la création d'observatoires nationaux de la santé mentale qui serviront à orienter des politiques économiques, sociales et sanitaires coordonnées, mais aussi à favoriser l'évolution vers une économie du bien-être plus durable, laissant entrevoir une amélioration de la santé mentale au sein de la population.

## Resumen

A pesar del aumento de la promoción y las inversiones en sistemas de salud mental en todo el mundo, los avances en la reducción de la prevalencia de los trastornos mentales han sido limitados. En este documento, sostenemos que para lograr avances significativos en la salud mental de la población es necesario abordar las fuentes fundamentales de la angustia compartida. Mediante una perspectiva sistémica, las estructuras y políticas económicas se identifican como la posible causa de los problemas de salud mental. Las ideologías neoliberales, que priorizan la optimización económica y el crecimiento continuo, contribuyen al fomento del individualismo, la inseguridad laboral, el aumento de las exigencias a los trabajadores, el estrés parental, la desconexión social y una gran variedad de manifestaciones bien reconocidas que perjudican la salud mental. Insistimos en la necesidad de que los investigadores y los defensores de la salud mental se impliquen cada vez más en el discurso de la política económica para atraer la atención sobre las implicaciones para la salud mental y el bienestar. Pedimos un cambio hacia una economía del bienestar para alinear mejor los intereses comerciales con el bienestar colectivo y la prosperidad social. Para movilizar a las comunidades en favor del cambio e influir en las políticas económicas con el fin de salvaguardar el bienestar, es necesaria la participación de personas que han padecido enfermedades mentales, profesionales e investigadores. Además, pedimos la creación de observatorios nacionales de bienestar mental que sirvan de base a las políticas sanitarias, sociales y económicas coordinadas y permitan la transición a una economía del bienestar más sostenible, que ofrezca perspectivas de progreso en los resultados de salud mental de la población.

## ملخص

على الرغم من زيادة الدعم والاستثمارات في أنظمة الصحة العقلية على مستوى العالم، فقد تم إحراز تقدم محدود في الحد من انتشار الاضطرابات العقلية. في هذه الورقة، نزعم أن التطورات ذات المغزى في الصحة العقلية للسكان تستلزم معالجة المصادر الأساسية للضيق المشترك. وباستخدام منظور النظم، يتم تمييز الهياكل والسياسات الاقتصادية باعتبارها السبب المحتمل لأسباب اعتلال الصحة العقلية. إن أيديولوجيات الليبرالية الجديدة، والتي تمنح الأولوية للتحسين الاقتصادي والنمو المستمر، تسهم في تعزيز النزعة الفردية، وانعدام الأمن الوظيفي، وزيادة الطلب على العمال، والضيق الأبوي، والانفصال الاجتماعي، ومجموعة واسعة من المظاهر المعروفة جيدًا بأنها تؤدي إلى تدهور الصحة العقلية. ونؤكد على حاجة الباحثين والمدافعين عن الصحة العقلية إلى المشاركة بشكل متزايد في خطاب السياسة الاقتصادية للفت الانتباه إلى الآثار المترتبة على الصحة العقلية والرفاهية. وندعو إلى التحول نحو اقتصاد الرفاهية من أجل مواءمة المصالح التجارية بشكل أفضل مع الرفاهية الجماعية والرخاء الاجتماعي. هناك حاجة إلى مشاركة الأفراد الذين عاشوا تجارب اعتلال الصحة العقلية، والممارسين، والباحثين، بهدف تعبئة المجتمعات من أجل التغيير والتأثير على السياسات الاقتصادية لحماية الرفاهية. بالإضافة إلى ذلك، ندعو إلى إنشاء مراصد وطنية للثروة العقلية بهدف توجيه السياسات الصحية والاجتماعية والاقتصادية المنسقة، وتحقيق الانتقال إلى اقتصاد رفاهية أكثر استدامة، ويقدم وعدًا بالتقدم في نتائج الصحة العقلية للسكان.

## 摘要

尽管已在全球范围内加大对心理健康系统的支持和投资，但在降低心理障碍患病率方面所取得的进展却十分有限。我们在本文中主张，若想在人口心理健康方面取得有意义的进步，就必须要解决导致共同痛苦的根本原因。从系统的角度分析，经济结构和政策被认为是导致心理健康状况不佳的潜在原因。新自由主义意识形态，优先考虑经济的最优化和持续增长，助长了倡导个人主义、工作无保障、对工人的需求日益增加、父母的养育压力、社会脱节以及各种被公认的有损心理健康的表现。我们强调心理健康研究人员和支持者需要更多地参与经济政策话语，从而引起人们对心理健康和幸福感的关注。我们呼吁发展幸福经济，将商业利益与集体福祉和社会繁荣更好地结合起来。需要有心理健康状况不佳经历的个人、从业人员和研究人员的参与，才能推动社区进行变革并影响经济政策，从而保障人们的福祉。此外，我们还呼吁建立国家精神财富观察站，为制定协调一致的卫生、社会和经济政策提供信息，并完成向更可持续的幸福经济的过渡，这样才有望在人口心理健康成果方面取得进展。

## Резюме

Несмотря на усиленную информационно-просветительскую деятельность и инвестиции в системы охраны психического здоровья во всем мире, прогресс в снижении распространенности психических расстройств незначителен. В этой статье авторы выдвигают тезис о том, что значимые достижения в области психического здоровья населения требуют устранения основных источников коллективного стресса. С точки зрения системного подхода экономические структуры и руководящие принципы определяются как возможные причины ухудшения психического здоровья. Идеология неолиберализма, которая на первое место ставит оптимизацию экономических показателей и непрерывный рост, способствует развитию индивидуализма, отсутствию гарантий занятости, повышению требований к работникам, родительскому стрессу, социальной изоляции и целому ряду проявлений, которые, как хорошо известно, подрывают психическое здоровье. В этой статье подчеркивается, что исследователи и защитники психического здоровья должны более активно участвовать в обсуждении экономической политики, чтобы привлечь внимание к последствиям для психического здоровья и благосостояния. Авторы призывают перейти к экономике благосостояния для лучшего согласования коммерческих интересов с коллективным благосостоянием и социальным благополучием. Для того чтобы сподвигнуть общественность на изменения и повлиять на экономическую политику ради обеспечения благосостояния, необходимо участие людей, имеющих опыт жизни с психическими заболеваниями, практикующих врачей и исследователей. Кроме того, авторы призывают к созданию национальных исследовательских центров по вопросам психического здоровья для разработки согласованной политики в области здравоохранения, социальной и экономической политики и перехода к более устойчивой экономике благосостояния, которая открывает перспективы для достижения прогресса в области психического здоровья населения.

## Introduction

Mental health systems are under-resourced, fragmented, inefficient and unable to meet the demands of the growing burden of mental and substance use disorders. Despite the intensive advocacy and action of leading global health and development organizations, little progress appears to have been made in reducing the prevalence of mental disorders since the 1990s.^1^ Even in high-income countries where investments in and access to mental health care have increased, prevalence is unchanged.^2^ A recent study indicates that the unchanged prevalence of mental disorders is likely due to increases in treatment provision being insufficient to offset a concurrent increase in the incidence of high to very high psychological distress driven by the economic and social environments in which we live.^3^

A broad range of social and environmental factors influence mental health. Such factors include adverse early life exposures, substance misuse, exposure to family and community violence, unemployment, financial insecurity, poverty, poor education quality, homelessness, inequality, racism, social exclusion, natural disasters, climate change, and other social and environmental factors that have unidirectional and bidirectional relationships with mental health and with each other in a complex causal web.^4^ In recognition of these important drivers of mental disorders, the World Health Organization’s (WHO) comprehensive *Mental Health Action Plan 2013–2030* calls for partnerships across health, education, employment, housing, social, private, judicial and other relevant sectors to deliver a comprehensive and coordinated response.^5^ Setting aside the challenges associated with investing sufficiently to address such a large number of social factors, we must question: to what extent are we missing an opportunity to understand and pursue root causes when we view all social determinants with equivalent importance?

In 2014, an article put forward an important question about the root causes of ill-health,^6^ pointing to key socioeconomic determinants such as education, income and wealth as the underlying causes of a wide range of health outcomes. Increasing attention is also being given to the commercial determinants of physical and mental health, that is, commercial sector influences including harmful products as well as marketing and employment practices, among others. Here we argue the pivotal role economic structures and policies play in shaping the commercial, social and environmental determinants of mental health, proposing economic forces as a cause of causes of mental illness. Additionally, we contend that addressing the burden of mental disorders is an important part of sound economic management.

## The economy as a root cause

Optimizing economic structures to ensure consistent growth in gross domestic product through increases in labour market flexibility, open markets and globalization has long been believed to be the path to achieving prosperity for all, allowing governments to afford investments in other crucial areas. However, prioritizing the optimization of one system above all others can have unintended consequences, destabilizing interconnected social, environmental and political systems and leading to a host of symptoms – the very symptoms we recognize to be the social determinants of health and well-being. Numerous authors have suggested that neoliberalism was oversold in its ambition to achieve an efficient, self-regulating economy.^7^ Moreover, neoliberalism favours values and approaches such as individualism, self-reliance, competitiveness, self-interest, expanding material consumption and limited government intervention or collective support, where the merits of human actions are assessed only by their market value.^8^ Such ideology absolves the individual of obligations to the collective, denies the human needs of social connection and erodes the social fabric of communities, undermining mental health.

### Economic structures 

While economic structures, appropriately designed, operated and managed have the potential to bring about prosperity, they can also lead to highly unequal distributions of information, technology, wealth and power.^9^ Inequality weakens individual agency, creates resentment, erodes social capital and trust, and increases crime rates.^10^^,^^11^ Inequality also contributes to political polarization, social unrest and erosion of community cohesion, as societies become divided over issues such as immigration, trade policies and wealth redistribution.^12^^,^^13^ Evidence suggests a negative feedback wherein inequalities undermine growth through the erosion of productive potential over the life course and across generations.^14^^,^^15^ Recent systematic reviews confirm an association between income inequality and poorer mental health, including a greater risk of depression in populations with higher income inequality relative to populations with lower inequality, and greater impacts among women and low-income groups.^16^^,^^17^ This evidence is of significant concern given that inequality within nations is growing for more than two thirds of the global population, particularly in high-income countries,^18^ a situation further exacerbated by the coronavirus disease 2019 (COVID-19) pandemic. The unequal distribution of productivity gains from expanding applications of generative artificial intelligence and associated market concentration and technological unemployment is likely to further widen inequalities.

### Globalization 

While globalization brings opportunity, particularly to emerging economies, diffusion of knowledge and sharing of natural resources that would otherwise be unevenly endowed, it has also led to cultural homogenization, income inequality, over-exploitation of natural resources, environmental destruction and climate change, which all have implications for mental health and well-being.^19^^–^^22^ For example, the spread of global brands, media and values, and the global movement of workforces can erode local traditions, customs, identities, and social and community support systems.^20^ In turn, these impacts can lead to feelings of displacement and loss among communities, as well as to a sense of cultural isolation and loneliness among individuals. Growing evidence exists that environmental factors such as air and water quality, exposure to plastics, noise and light pollution, food quality and security, access to green space and the effects of climate change affect mental health and well-being.^23^^–^^26^

### Economic policies

Economic policies also play an important role in shaping the social environment that influences mental health.^27^ Emphasis on sustained growth in labour productivity can reduce worker well-being through increases in job demands, alienation, anxiety, work-related family conflict and burnout.^28^ Industrial relations reforms, found in almost all advanced nations, are aimed at fostering business flexibility, creating jobs and stimulating economic growth. However, these reforms can result in labour markets characterized by insecure, fixed-term, temporary and casualized work, thereby increasing vulnerability.^29^^–^^31^ Job insecurity can have a detrimental effect on work satisfaction and mental well-being, and can increase depressive symptoms.^32^ Precarious employment arrangements can also undermine the economic security of households; disrupt social connections; and increase isolation,^33^ marital tension, parental stress,^34^ and rates of domestic violence^35^ and child abuse and neglect.^36^ Adverse experiences early in life can have profound effects on mental health and development, increasing rates of psychological distress, substance misuse, physical health issues, behavioural challenges and suicidal behaviour,^37^^–^^40^ and can perpetuate intergenerational disadvantage. The negative social and mental health manifestations of neoliberal policies reflect the concept of anomie^41^ – a breakdown of the social values, norms, opportunities and ties that keep complex societies together, give people a sense of belonging, of hope for the fulfilment of their ambitions and needs, and support during periods of disruption and grief. Anomie can lead to alienation, distress, despair and deviant behaviour.^41^

Unless policy-makers address the root cause of the drivers of mental illness – that is, economic structures and policies – progress towards reductions in the adverse social determinants of mental health may not be achievable or sustainable. Likewise, considerable unmet needs for mental health services will likely remain, even if countries increase mental health expenditure. Healthy, functional economies are essential to the well-being of nations, communities and individuals, and vice versa. As highlighted by the WHO Council on the Economics for Health for All,^42^ it is critical that nations take steps to restructure their economies in ways that support both the mental and material dimensions of well-being and human flourishing. Economic policies and initiatives that offer promise in strengthening the mental health and mental wealth^43^ of nations have been discussed in another publication.^44^ A well-being economy seeks to better align and balance the interests of collective well-being and social prosperity with traditional economic and commercial interests. This approach seeks to realize a more inclusive, equitable, sustainable and resilient society.

## Beyond disciplinary boundaries

While the challenges of seeking to reform the prevailing global economic system seem insurmountable, movement towards a well-being economy is gaining significant momentum as economists, policy-makers and the public are recognizing the limitations of traditional economic models, including their detrimental impacts on society, the environment, health and mental well-being. The establishment of mental wealth observatories (Box 1) that would bring together granular health, social and economic data, and scientists working across disciplines such as economics, social science, psychology, psychiatry, mathematics, biostatistics, business and the science of complex systems is critical to the transition to a well-being economy. Through six integrated streams of activities (Box 1), mental wealth observatories provide essential national infrastructure to coordinate transdisciplinary actions. Priorities would include (i) regular reporting on the strength of the well-being economy, that is, the mental wealth of nations (Stream 1);^43^ (ii) generating the transdisciplinary science needed to inform systemic reforms and coordinated policies across economic, environmental, health and social sectors (Streams 2, 3, 4); and (iii) engaging in the communication and diplomacy needed to achieve national and international cooperation in transitioning to a well-being economy (Streams 5, 6). A policy memo provides a more detailed blueprint for such activities.^46^

Box 1 **Blueprint for a national mental wealth observatory**

The aim of national mental wealth observatories is to provide the transdisciplinary data and science needed to inform systemic actions to transition to a well-being economy, building multisystem resilience, human flourishing and national prosperity. The observatories will have six overlapping streams of activity.

***Stream 1: Mental wealth – measuring and monitoring the strength of a well-being economy***

While several research communities and nations are embracing well-being economy frameworks and tracking progress against a broad range of indicators of individual and societal well-being, an overarching measure of progress is needed. Without it, GDP will remain the privileged indicator that policy levers are trained on. Therefore, this stream is focused on the further evolution of GDP to be a more holistic topline indicator of the strength of a well-being economy. This indicator is called mental wealth.

Mental wealth is a measure that extends the boundary of GDP to include social production (the value of unpaid social contributions).^43^ Social production is the glue that holds complex societies together. Social contributions foster community well-being, support economic productivity, improve environmental well-being and provide nations with surge capacity to respond to crises. Mental wealth reflects the health of the economy and the health of society and hence is the measure of the strength of a well-being economy. Mental wealth elevates mental health’s importance as a policy focus, and its standardized universal measurement will inform policy and advocacy for change.

***Stream 2: Complex systems modelling and simulation***

This stream is focused on advancing from rudimentary analytic and decision support tools to harness complex systems modelling and simulation that will inform greater alignment of policies across economic, social and health systems and enhance the mental wealth of nations. Further information is provided in sections V and VI of *Measuring, modelling, and forecasting the mental wealth of nations*.^45^

Developing systems models requires the integration of scientific theory, with best available research evidence, and diverse data sources in a way that allows decision-makers to evaluate alternative policies and initiatives, or ask resource allocation questions in a safe virtual environment before implementing them in the real world. As new evidence and data become available, models are updated and/or refined, becoming more robust over time and offering significant value as long-term decision support assets.

***Stream 3: Strengthening transdisciplinary data ecosystems***

This stream will strengthen transdisciplinary data ecosystems by harnessing advances in technology, passive and/or sentinel surveillance, and will provide intelligence to inform coordinated cross-sectoral policy and planning.

This stream will also support early detection and rapid response to system stress, and inform both Stream 2 modelling and Stream 4 Brain Capital research programme. This programme will include the establishment of a brain capital dashboard and ongoing monitoring of brain capital indicators. In addition, the Mental Wealth Initiative at the University of Sydney is developing innovative protocols. For example, a protocol for wastewater monitoring of stress hormones like cortisol and cortisone is under development to gain near-real-time insights into community stress and inform rapid deployment of resources and/or infrastructure to support communities through challenging times and prevent social decline before it becomes entrenched.

***Stream 4: Brain Capital research programme***

This research programme will harness advanced research technologies to answer priority questions of global or national relevance. Such questions may include (i) what are the likely impacts of artificial intelligence on the diffusion of productivity gains, wealth and well-being? (ii) what are the projected impacts of early childhood education and care on school readiness, workforce participation and family income? (iii) what is the relationship between social capital infrastructure investment, social connectedness and mental health in young people? (iv) how is artificial intelligence changing the nature of work, well-being and productivity? (v) what is the optimal balance of digital technologies and human workforces needed to scale mental health and social care to meet demand? and (vi) how can employers and educators work together to create workforces and workplaces that are adaptable to changing circumstances by mastering quality, transferable vocational skills?

***Stream 5: Knowledge translation***

Shifting entrenched economic narratives and frameworks will require more than the efforts of economists alone. This stream will focus on transdisciplinary policy advocacy, knowledge translation, and public communications because stable transition to a well-being economy will require broad scientific, policy and public support as well as better cooperation between public and private sectors.

***Stream 6: Brain health and science diplomacy***

Nothing less than an ambitious, innovative, transdisciplinary and coordinated transnational research agenda is needed to enable the transition to a well-being economy. The open sharing of insights, tools and metrics across global agencies is needed to elevate the importance of mental health as a policy focus and inform policy and advocacy efforts and momentum for change. Therefore, this stream will focus on building bridges between countries through a universal appreciation of the importance of the integrity of the social fabric of nations for a nation’s stability and resilience. Science diplomacy will also be important in facilitating the sharing of knowledge and innovations across borders, as well as for fostering international cooperation in transitioning to a well-being economy.

GDP: gross domestic product. Source: Occhipinti J, Eyre HA, Hynes W, 2023.^46^

Addressing the root causes of common mental disorders in addition to the severe underinvestment in mental health systems and system fragmentation is crucial. Substantial improvements in mental health are unlikely if the economic structures and policies that have undermined the social fabric and well-being of nations continue unchallenged, and hence this should be a focus of future national mental health action plans. An ambitious, innovative, transdisciplinary and coordinated transnational research agenda is needed to build momentum for change and enable the transition to a well-being economy.
